# Association between total cholesterol and total bone mineral density in US adults: National Health and Nutrition Examination Survey (NHANES), 2011–2018

**DOI:** 10.1186/s13018-022-03485-8

**Published:** 2023-01-16

**Authors:** Li cao, Wei Wu, Xiangyu Deng, Haoyu Guo, Feifei Pu, Zengwu Shao

**Affiliations:** Department of Orthopaedic, Union Hospital, Tongji Medical College, Huazhong University of Science and Technology, Wuhan, 430022 People’s Republic of China

**Keywords:** Bone mineral density, Cholesterol, Osteopenia, Osteoporosis

## Abstract

**Background:**

Accumulated evidence indicates that cholesterol is offensive to bone metabolism. Therefore, we examined the real-world study among total cholesterol and total bone mineral density (BMD). We investigated the relationship between total cholesterol and total BMD among 10,039 US participants aged 20–59 years old over the period 2011–2018 from the NHANES.

**Methods:**

To analyze the relationship among total cholesterol and total BMD, multivariate linear regression models were used. Fitted smoothing curves, generalized additive models, and threshold effect analysis were also conducted.

**Results:**

After adjusting for additional covariates, weighted multivariable linear regression models indicated total cholesterol concentration levels exhibited a negative relationship with total BMD, particularly among participants aged 20–29 years. Concerning subgroup analysis, stratified by gender, race/ethnicity and age group, the negative correlation of total cholesterol with total BMD dwelled in both female and male as well as in whites and other races (including Hispanic and Multi-Racial), but not in non-Hispanic blacks and Mexican American. In other races, this relationship presented a nonlinear association (inflection point: 6.7 mmol/L) with a *U*-shaped curve. Among participants aged 40 to 49 years, this relationship also followed a nonlinear association (inflection point: 5.84 mmol/L), indicating a saturation effect. Moreover, the three types of diabetes status were found to have negative, *U*-shaped, and positive relationships. In participants with borderline diabetes status, the relationship of total cholesterol with total BMD was a *U*-shaped curve (inflection point: 4.65 mmol/L).

**Conclusions:**

For US young adults (20–29 years old), our study revealed a negative relationship between total cholesterol and total BMD. This association followed a *U*-shaped curve (inflection point: 4.65 mmol/L) in borderline diabetes status participants, a saturation curve (inflection point: 5.84 mmol/L) in participants aged 40–49 years and a nonlinear curve (inflection point: 6.7 mmol/L) in other races (including Hispanic and Multi-Racial). Therefore, keeping total cholesterol concentration at a reasonable level for young adults and diabetic population might be an approach to prevent osteoporosis or osteopenia.

## Background

Cholesterol is a major lipid element of human organisms and plays a pivotal role in bile acid metabolism, steroid hormone synthesis, and bone cell metabolism [1]. In the past decades, serum cholesterol is considered being risky in a variety of diseases such as atherosclerosis, Parkinson’s disease, and osteoarthritis [2–4]. Recently, cholesterol toxicity, which globally influences organs, was put forward due to its activation of inflammation, endoplasmic reticulum stress, and mitochondrial dysfunction via blood circulation [5]. Some retrospective studies show that low-density lipoprotein (LDL-C) level of total cholesterol is negatively correlated with lumber BMD in women, while high-density lipoprotein (HDL-C) which is regarded to be favorable to health is positive [6–8]. Total cholesterol could be related to BMD, but the results reported from different research were disparate [9–11]. Therefore, the latest evidence about total cholesterol level associated with total BMD in adults is needed from a holistic perspective.

Osteoporosis, defined as a high rates chronic disease featured with reduced BMD of whole body, affects 9.9 million Americans [12, 13]. Concurrently, as the population ages and imbalanced dietary intake, the osteoporosis population is set to increase [14, 15]. Apart from senile osteoporosis or postmenopausal osteoporosis, adult osteopenia in men or women is a concern and maybe a stage to intervene [16–18]. According to a group of studies, the risk of osteoporotic fracture for males and females is roughly 30% and 50% in a lifetime, respectively [19–21]. Exploring influencing factors like total cholesterol may help to intervene early in adults. Some risk factors are considered to be related to osteoporosis, such as increasing age, white race, female sex, lipid metabolism and heredity in older people, but not in adults [22, 23]. Therefore, it is necessary to investigate the relationship between total cholesterol for adults aged 20 to 59 years to discover more valuable information.

Total cholesterol level was important in the evaluation of cardiovascular disease in adults and associated with mortality, but rarely noticed in total BMD [24, 25]. Accordingly, to explore if the total cholesterol could influence BMD and find an appropriate total cholesterol level avoiding osteoporosis, we used the 10,039 latest participants from NHANES 2011–2018. We also stratified participants by gender, race and age to determine specific effect on a nationally representative participates aged 20–59 years.

## Materials and methods

### Ethics statement

The study was approved by the National Center for Health Statistics' Ethics Review Board (https://www.cdc.gov/nchs/nhanes/irba98.htm), and informed consent was obtained from all NHANES participants for their data to be adopted in further study.

### Study participants and inclusion criteria

**Fig. 1:**
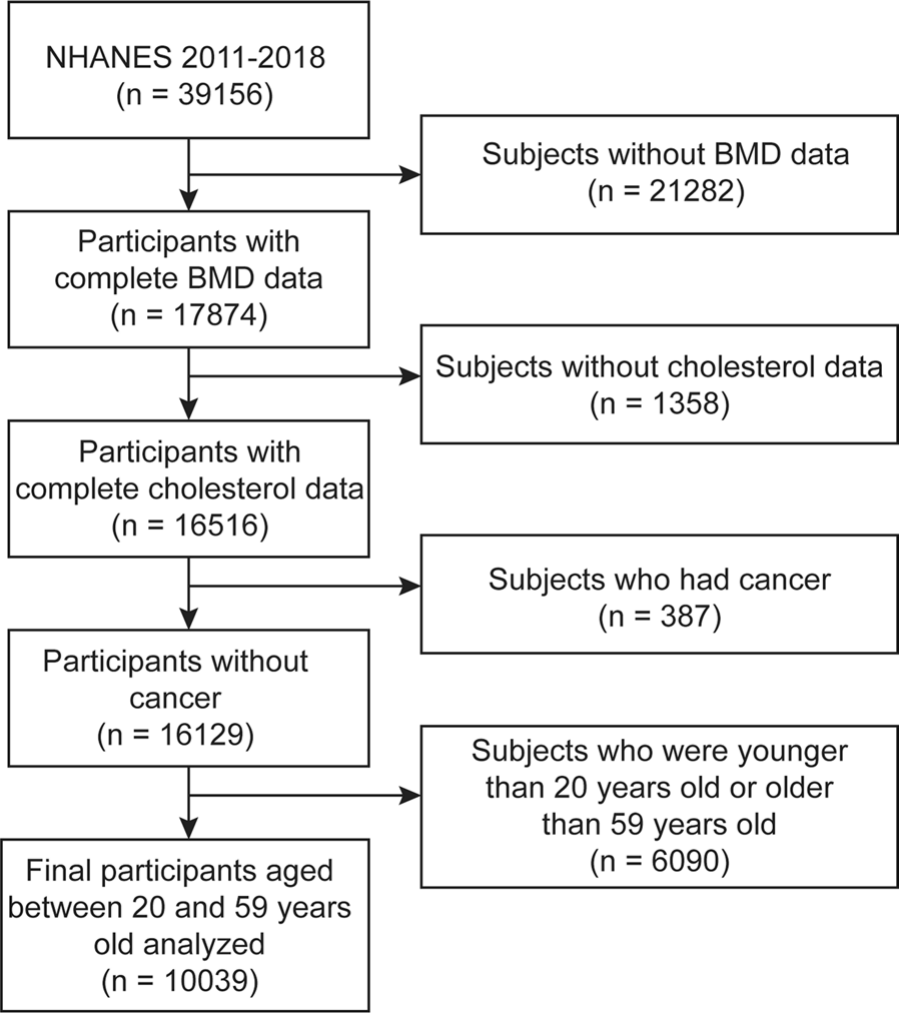
Flowchart of participants selection. *NHANES* National Health and Nutrition Examination Survey; *BMD* Bone mineral density

The NHANES is an ongoing representative epidemiological study in the USA that intends to survey multifarious evidence about the nutrition and health statistics utilizing a multistage, complex, probability sampling design. Our analysis was based on data from 2011 to 2018, which represents four cycles of the NHANES datasets. All data of subjects was collected from the NHANES database, which is part of the U.S. Centers for Disease Control and Prevention. The inclusion criteria were: (i) individuals who had accessible total BMD data; (ii) individuals who had accessible total cholesterol data; and (iii) individuals aged 20 to 59 years old. Moreover, the subjects with cancer were excluded from the study. Of a total 39,156 participants, we excluded 21,282 subjects with missing total BMD data, 1,358 participants with missing total cholesterol data, 387 subjects with cancer diagnoses, and 6,090 subjects younger than 20 years or older than 59 years. Finally, 10,039 participants were used in the research (Fig. 1).

### Variables

**Fig. 2:**
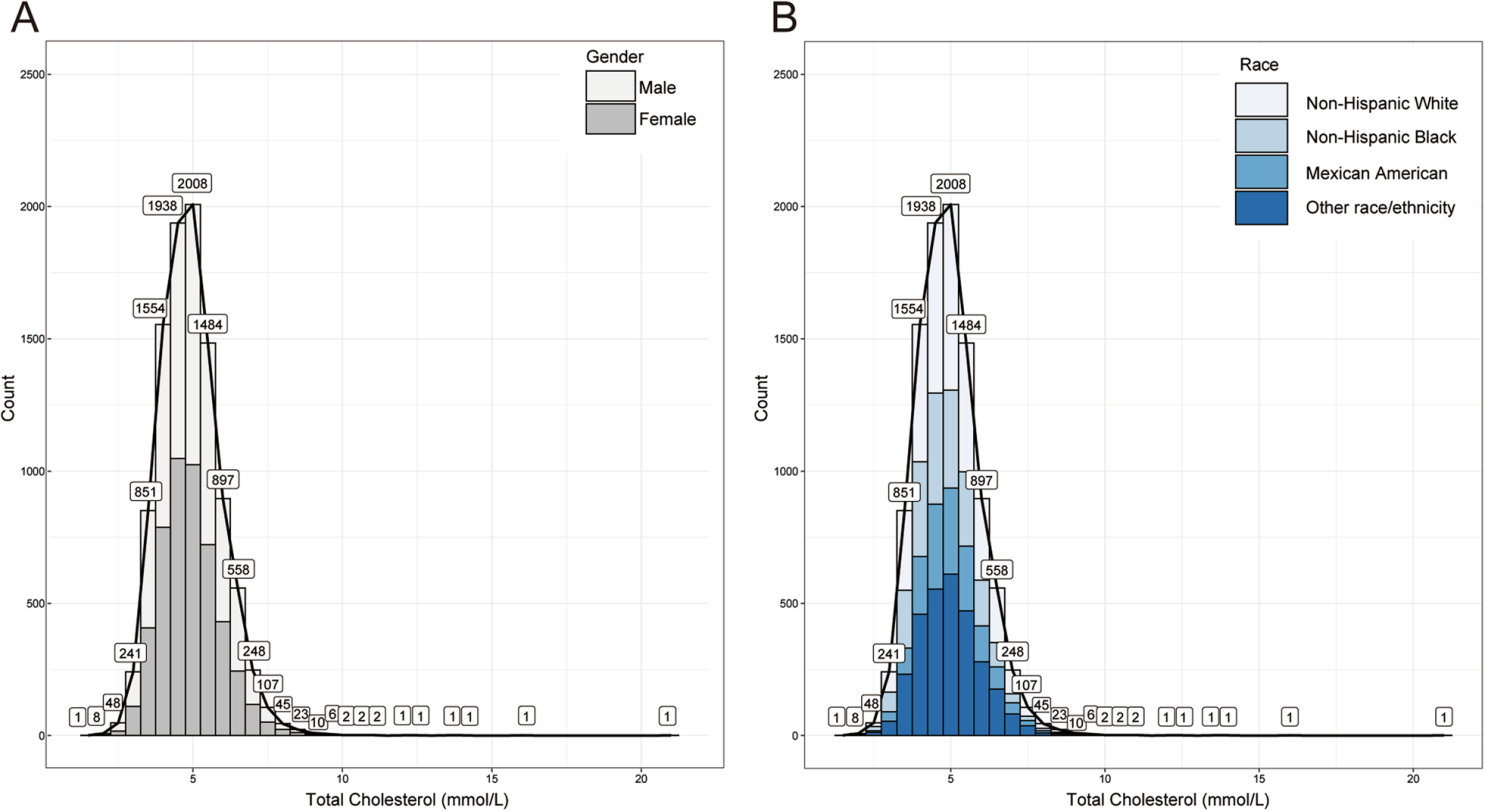
Frequency histogram (bin = 0.5) of total cholesterol. **A **Grouped by gender; **B ** Grouped by race/ ethnicity

Total cholesterol as the exposure variable was measured via a serum sample by Roche Cobas 6000, which is an enzymatic method where esterified cholesterol is converted to cholesterol by cholesterol esterase and then acted upon by cholesterol oxidase to produce cholest-4-en-3-one and hydrogen peroxide. The hydrogen peroxide then reacts known as the Trinder reaction with 4-aminophenazone in the presence of peroxidase to produce a colored product that is measured at 505 nm (secondary wavelength = 700 nm). Total BMD testing was performed by Dual-energy X-ray absorptiometry (DEXA) by Hologic QDR 4500A bone densitometers and Apex software (version: 3.2) throughout by certified radiology technologists. For categorical variables, the study included the following covariates: gender, race/ethnicity, educational level, moderate activity, smoking, marital status, high blood pressure status, and diabetes status. The study included ratio of family income-to-poverty, waist circumference, BMI, calcium intake, serum calcium, alcohol consumption, ALT, AST, blood urea nitrogen, serum phosphorus, γ-glutamyl transferase, lactate dehydrogenase, serum uric acid and triglycerides as continuous covariates. The categories "other Hispanic" and "other multiracial race" from the original NHANES were assigned to the variable "other race/ethnicity" to lower the bias of sample quantity. The NHANES program (https://www.cdc.gov/nchs/nhanes/) describes more detailed information about how the variables were measured (Fig. 2).

### Statistical analysis

We used weighted and variance estimation analysis to explain the significant differences in our data sets. Weighted multivariate logistic regression models were applied aiming to evaluate the link between total cholesterol and total BMD. We applied weighted multivariate linear regression models and weighted *χ*2 tests to assess the distinction among each group for the categorical and continuous variables. To subgroup analysis, stratified multivariate regression analysis was also made. Moreover, nonlinear associations between total cholesterol and total BMD were also discussed via smooth fitting curves and generalized additive models. For nonlinear models, if the nonlinearity was detected, then the two-piecewise linear regression models was performed on both parts of the inflection point using a recursive algorithm. All analyses were performed using EmpowerStats (version: 2.0, X&Y Solutions, Inc., Boston, MA. http://www.empowerstats.com) and *R* software (version: 4.0.3, http://www.R-project.org), with a *P* value < 0.05 considered significant, and the frequency histogram of total cholesterol was completed via ggplot2 package in *R*.

## Results

### Participants characteristics

The weighted characteristics of participants were separated into quartiles based on total cholesterol concentration (Q1: 1.50–4.20 mmol/L; Q2: 4.21–4.80 mmol/L; Q3: 4.81–5.51 mmol/L; and Q4: 5.52–21.02 mmol/L), as displayed in Table 1. A total of 10,039 participants aged 20–59 years old were covered in our study. Our study showed significant differences in baseline characteristics between the total cholesterol concentration quartiles (Table 1). Participants from the Q4 total cholesterol quartile were intended to be males, non-Hispanic whites, with greater values of BMI (29.39 ± 5.89 kg/m^2^), waist circumference, alcohol consumption, blood urea nitrogen, ALT, AST, serum phosphorus, serum calcium, *γ*-glutamyl transferase, lactate dehydrogenase, serum uric acid, triglycerides, and ratio of family income-to-poverty and lower total BMD, moderate activity, and low calcium intake compared to the other level groups.

**Table 1:** Weighted characteristics of the study population based on total cholesterol quartiles

Total cholesterol (mmol/L)	All	Q1 (1.50–4.20)	Q2 (4.21–4.80)	Q3 (4.81–5.51)	Q4 (5.52–21.02)	*P* value
Age (years, mean ± SD)	39.1475 ± 11.6236	34.3632 ± 11.5422	37.8907 ± 11.3880	40.0022 ± 11.0128	43.9945 ± 10.4141	< 0.001
Race/ethnicity (*n*, %)						< 0.001
Non-Hispanic White	60.6415	58.3164	60.1266	60.6492	63.3028	
Non-Hispanic Black	11.2247	14.3237	11.4634	9.9549	9.3376	
Mexican American	10.8656	10.1864	11.2935	11.8468	10.1315	
Other race/ethnicity^a^	17.2681	17.1735	17.1165	17.5491	17.2280	
Gender (n, %)						< 0.001
Male	50.5703	52.9875	47.5757	49.0817	52.6419	
Female	49.4297	47.0125	52.4243	50.9183	47.3581	
Smoked at least 100 cigarettes in life (n, %)						< 0.001
Yes	40.3948	36.4892	37.6179	41.3839	45.7500	
No	59.6052	63.5108	62.3821	58.6161	54.2500	
Education Level (*n*, %)						0.0222
Less than high school	13.0264	11.7858	14.5282	12.4560	13.2998	
High school	21.8284	22.9680	20.2915	21.2498	22.8050	
More than high school	65.1452	65.2462	65.1803	66.2941	63.8952	
Marital status (*n*, %)						< 0.001
Married	62.3723	56.3314	60.5319	65.6445	66.5949	
Not married	37.6277	43.6686	39.4681	34.3555	33.4051	
High blood pressure (*n*, %)						< 0.001
Yes	21.9384	18.6004	19.0398	23.9792	25.8529	
No	78.0616	81.3996	80.9602	76.0208	74.1471	
Diabetes (n, %)						< 0.001
Yes	5.6877	7.6649	4.7825	4.7758	5.5993	
No	92.6495	90.5706	93.6912	94.2886	91.9918	
Borderline	1.6628	1.7645	1.5262	0.9356	2.4090	
Moderate activity (*n*, %)						0.0893
Yes	43.6194	45.8512	43.0304	42.7664	42.9316	
No	56.3806	54.1488	56.9696	57.2336	57.0684	
Ratio of family income-to-poverty	2.8874 ± 1.6253	2.6719 ± 1.5960	2.8465 ± 1.6322	2.9634 ± 1.6149	3.0542 ± 1.6317	< 0.001
BMI (kg/m^2^, mean ± SD)	28.8526 ± 6.7012	28.0736 ± 7.1665	28.6352 ± 6.9363	29.2626 ± 6.7060	29.3896 ± 5.8874	< 0.001
Waist circumference (cm, mean ± SD)	97.6414 ± 16.1154	95.0032 ± 17.5097	96.7701 ± 16.9270	98.6266 ± 15.5517	99.9837 ± 13.9230	< 0.001
ALT (IU/L, mean ± SD)	26.0021 ± 19.4809	23.4796 ± 17.3589	24.7621 ± 17.5185	26.2276 ± 20.2821	29.3324 ± 21.7419	< 0.001
AST (IU/L, mean ± SD)	25.1347 ± 17.3020	24.2468 ± 22.0443	24.3868 ± 13.8280	25.0357 ± 15.4333	26.7806 ± 16.8691	< 0.001
Blood urea nitrogen (mmol/L, mean ± SD)	4.5516 ± 1.5137	4.4731 ± 1.6319	4.4469 ± 1.4514	4.5913 ± 1.4617	4.6867 ± 1.4954	< 0.001
Serum Calcium (mmol/L, mean ± SD)	2.3430 ± 0.0871	2.3321 ± 0.0884	2.3315 ± 0.0808	2.3430 ± 0.0833	2.3642 ± 0.0911	< 0.001
Serum Phosphorus (mmol/L, mean ± SD)	1.2025 ± 0.1804	1.1977 ± 0.1880	1.1952 ± 0.1739	1.2026 ± 0.1774	1.2138 ± 0.1817	0.0011
γ-glutamyl transferase (IU/L, mean ± SD)	28.0757 ± 43.6617	21.6725 ± 25.0934	25.2668 ± 34.6625	27.8617 ± 33.8083	36.9717 ± 66.3157	< 0.001
Lactate dehydrogenase (IU/L, mean ± SD)	129.7656 ± 28.6577	126.0251 ± 29.4127	128.6559 ± 28.2408	130.8497 ± 27.1949	133.2715 ± 29.2428	< 0.001
Serum uric acid (umol/L, mean ± SD)	316.7172 ± 80.9951	307.3026 ± 78.3826	309.9944 ± 78.4733	318.2396 ± 82.0241	330.4958 ± 82.7881	< 0.001
Triglycerides (mmol/L, mean ± SD)	1.6841 ± 1.5303	1.1937 ± 0.8179	1.3967 ± 0.8807	1.7269 ± 1.1597	2.3771 ± 2.3662	< 0.001
Calcium intake (g, mean ± SD)	995.6707 ± 587.4266	1016.9967 ± 631.5901	1020.7488 ± 616.8048	976.4544 ± 547.3698	970.3876 ± 550.5259	0.0016
Alcohol consumption (g, mean ± SD)	13.1840 ± 32.2826	10.5123 ± 28.5117	11.8214 ± 29.5059	14.0598 ± 33.0252	16.1365 ± 36.8261	< 0.001
Total BMD (g/cm^2^, mean ± SD)	1.1154 ± 0.1058	1.1252 ± 0.1059	1.1181 ± 0.1054	1.1138 ± 0.1034	1.1052 ± 0.1075	< 0.001

*ALT* Alanine aminotransferase, *AST* Aspartate aminotransferase, *BMI* Body mass index, *SD* Standard deviation, *n* Numbers of subjects, %, weighted percentage, *BMD* bone mineral density; For continuous variables: the *P* value was calculated by the weighted linear regression model. For categorical variables: the *P* value was calculated by the weighted chi-square test

^a^Other race/ethnicity included Hispanic and multiracial participants

**Fig. 3:**
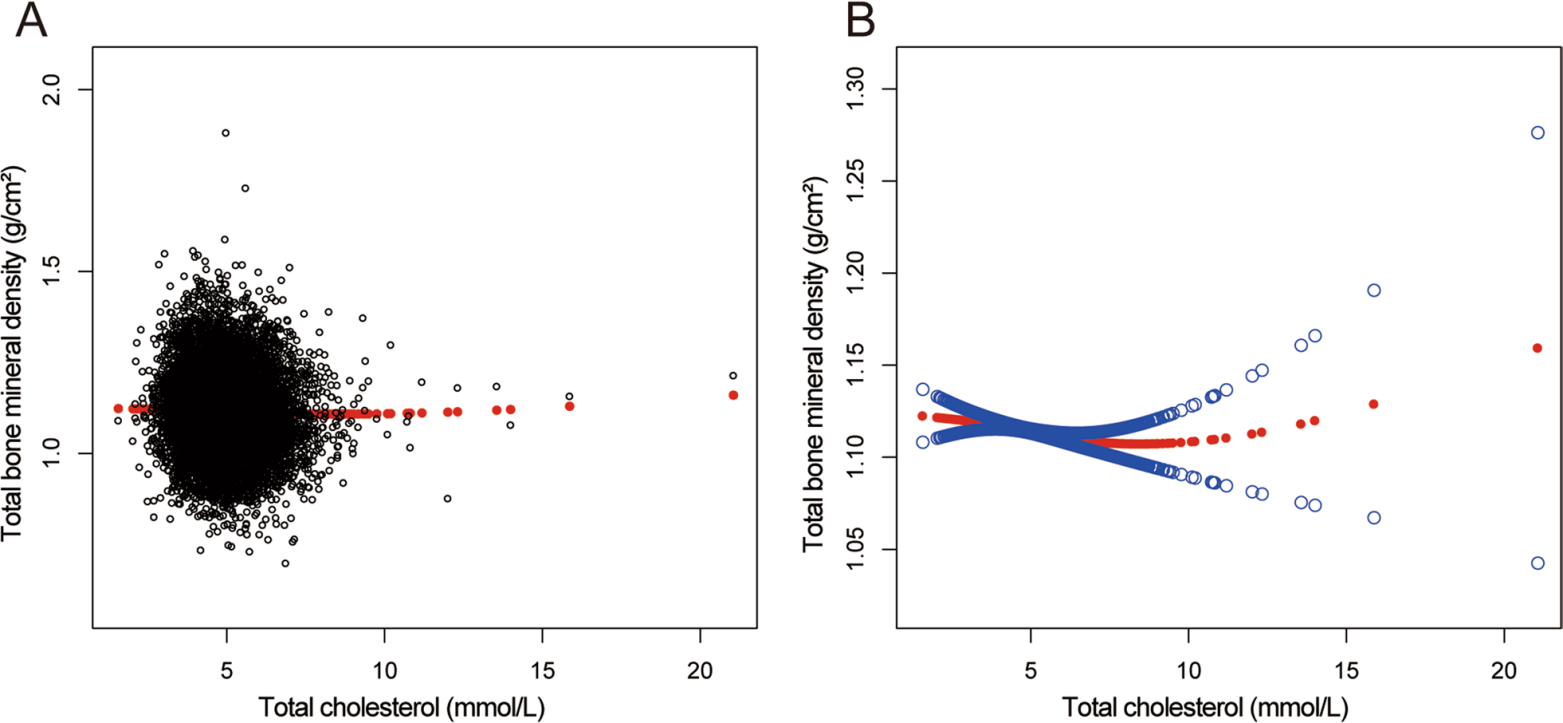
The relationship between total cholesterol and total bone mineral density. **A** Each black hollow point exhibits one participant. **B** Solid red line illustrates the fitted smooth curve among variables. Two blue bands illustrate the 95% confidence interval of the fit. Gender, age, race/ethnicity, education level, marital status, ratio of family income-to-poverty, serum phosphorus, blood urea nitrogen, *γ*-glutamyl transferase, ALT, AST, lactate dehydrogenase, serum uric acid, triglycerides, BMI, waist circumference, calcium intake, serum calcium, alcohol consumption, high blood pressure, moderate activity, diabetes and smoking were adjusted

Overall, the mean age of participants was 39.15 ± 11.62 years, the male (50.57%) and female (49.43%) were almost equal, and non-Hispanic whites account for 60.64%. Most of participates had an above high marriage rate (62.37%), high school education level (65.15%) and the ratio of family income-to-poverty was 2.89 ± 1.63. The participants had an educational level over high school, high blood pressure, and diabetes, which accounted for 65.15%, 21.94%, and 5.69%, respectively. Moreover, the distribution of total cholesterol categorized by gender and race is presented in Fig. 3. The specific results and other baseline characteristics are displayed in Table 1.

### Relationship between total cholesterol and total BMD

The multivariate regression analysis results are presented in Table 2. When no covariates are adjusted, total cholesterol was negatively associated with total BMD in Model 1 (*β* = − 0.0070, 95%CI − 0.0090 to − 0.0051, *P* < 0.001). After covariates were adjusted, this negative relationship was still present in Model 2 (*β* = − 0.0038, 95%CI − 0.0058 to − 0.0019, *P* < 0.001) and Model 3 (*β* = − 0.0036, 95%CI − 0.0057 to − 0.0015, *P* < 0.001). Participants of the highest total cholesterol quartile (Q4) had a 0.0109 g/cm^2^ lower total BMD than those in the base total cholesterol quartile after total cholesterol had been transformed from a continuing variable to a quartile’s variable.

**Table 2:** Association between total cholesterol (mmol/L) and total bone mineral density (g/cm^2^)

	Model 1, *β* (95%, CI)	Model 2, *β* (95%, CI)	Model 3, *β* (95%, CI)
	*P* value	*P* value	*P* value
Serum cholesterol (mmol/L)	− 0.0070 (− 0.0090, − 0.0051)	− 0.0038 (− 0.0058, − 0.0019)	− 0.0036 (− 0.0057, − 0.0015)
	< 0.001	< 0.001	< 0.001
Quintiles of cholesterol			
Lowest quintiles (0.15–4.23 mmol/L)	Reference	Reference	Reference
Q2(1.50–4.20 mmol/L)	− 0.0072 (− 0.0131, − 0.0013)	0.0005 (− 0.0050, 0.0059)	− 0.0001 (− 0.0055, 0.0053)
Q3(4.81–5.51 mmol/L)	− 0.0114 (− 0.0173, − 0.0055)	− 0.0025 (− 0.0080, 0.0030)	− 0.0040 (− 0.0095, 0.0015)
Q4(5.52–21.02 mmol/L)	− 0.0201 (− 0.0259, − 0.0142)	− 0.0112 (− 0.0169, − 0.0056)	− 0.0109 (− 0.0168, − 0.0050)
*P* for trend	< 0.001	< 0.001	< 0.001
Stratified by gender			
Men	− 0.0053 (− 0.0081, -0.0025)	− 0.0030 (− 0.0058, − 0.0002)	− 0.0037 (− 0.0067, − 0.0006)
	< 0.001	0.0198	0.020
Women	− 0.0086 (− 0.0112, − 0.0061)	− 0.0041 (− 0.0068, − 0.0015)	− 0.0037 (− 0.0065, − 0.0008)
	< 0.001	0.002	0.011
Stratified by race/ethnicity			
Non-Hispanic White	− 0.0065 (− 0.0098, − 0.0032)	− 0.0044 (− 0.0076, − 0.0011)	− 0.0040 (− 0.0075, − 0.0004)
	< 0.001	0.009	0.028
Non-Hispanic Black	− 0.0061 (− 0.0111, − 0.0012)	− 0.0012 (− 0.0060, 0.0037)	− 0.0005 (− 0.0056, 0.0046)
	0.014	0.632	0.844
Mexican American	− 0.0014 (− 0.0062, 0.0035)	− 0.0024 (− 0.0072, 0.0024)	− 0.0028 (− 0.0081, 0.0026)
	0.584	0.329	0.316
Other race/ethnicity	− 0.0064 (− 0.0100, − 0.0028)	− 0.0045 (− 0.0080, − 0.0010)	− 0.0047 (− 0.0086, − 0.0008)
	< 0.001	0.0126	0.017
Stratified by age			
20 ≤ Age < 30	− 0.0033 (− 0.0079, 0.0012)	− 0.0017 (− 0.0059, 0.0025)	− 0.0040 (− 0.0086, 0.0006)
	0.152	0.437	0.086
30 ≤ Age < 40	− 0.0012 (− 0.0054, 0.0030)	− 0.0046 (− 0.0086, − 0.0007)	− 0.0030 (− 0.0073, 0.0013)
	0.575	0.020	0.168
40 ≤ Age < 50	− 0.0008 (− 0.0045, 0.0030)	− 0.0008 (− 0.0043, 0.0027)	− 0.0006 (− 0.0045, 0.0033)
	0.689	0.6617	0.770
50 ≤ Age < 60	− 0.0166 (− 0.0208, − 0.0124)	− 0.0049 (− 0.0068, − 0.0030)	− 0.0048 (− 0.0069, − 0.0028)
	< 0.001	< 0.001	< 0.001

Model 1: No covariates were adjusted. Model 2: Age, gender and race/ethnicity were adjusted. Model 3: Age, gender, race/ethnicity, ratio of family income-to-poverty, education level, marital status, blood urea nitrogen, serum phosphorus, *γ*-glutamyl transferase, ALT, AST, lactate dehydrogenase, serum uric acid, triglycerides, BMI, waist circumference, serum calcium, calcium intake, alcohol consumption, high blood pressure, moderate activity, diabetes and smoking were adjusted. In the subset analysis stratified by sex, race/ethnicity, and age, the model is not adjusted for sex, race/ethnicity and age, respectively

### Subgroup analysis

Subgroup analyses, stratified by gender, age, and, race/ethnicity, are presented in Table 2. The adverse connection of total cholesterol with total BMD remained in both females (β = − 0.0037, 95%CI − 0.0065 to − 0.0008, *P* = 0.011) and males (*β* = − 0.0037, 95%CI − 0.0067 to -0.0006, *P* = 0.020), as well as in non-Hispanic white (*β* = − 0.0040, 95%CI − 0.0075 to − 0.0004, *P* = 0.028) and other races (β = − 0.0047, 95%CI − 0.0086 to − 0.0008, *P* = 0.017), but not in Mexican Americans and non-Hispanic blacks. Moreover, the significant negative correlation still existed in participants aged 50–59 years (*β* = − 0.0048, 95%CI − 0.0069 to − 0.0028, *P* < 0.001).

**Fig. 4:**
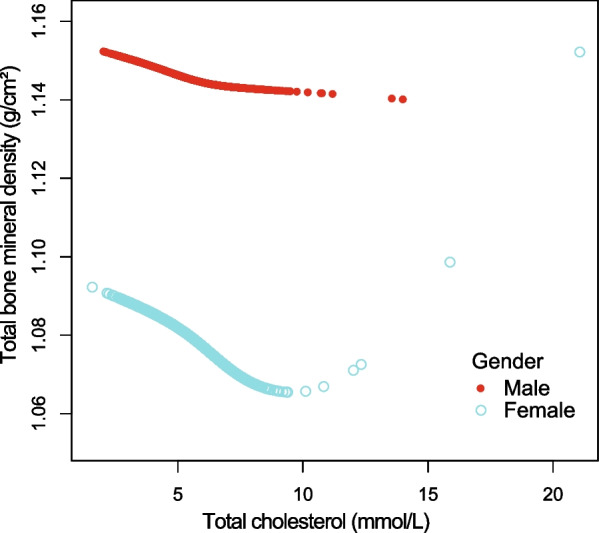
The relationship between total cholesterol and total bone mineral density stratified by gender. Age, gender, race/ethnicity, ratio of family income-to-poverty, education level, marital status, blood urea nitrogen, serum phosphorus, *γ*-glutamyl transferase, ALT, AST, lactate dehydrogenase, serum uric acid, triglycerides, BMI, waist circumference, calcium intake, serum calcium, alcohol consumption, high blood pressure, moderate activity, diabetes, and smoking were adjusted

**Fig. 5:**
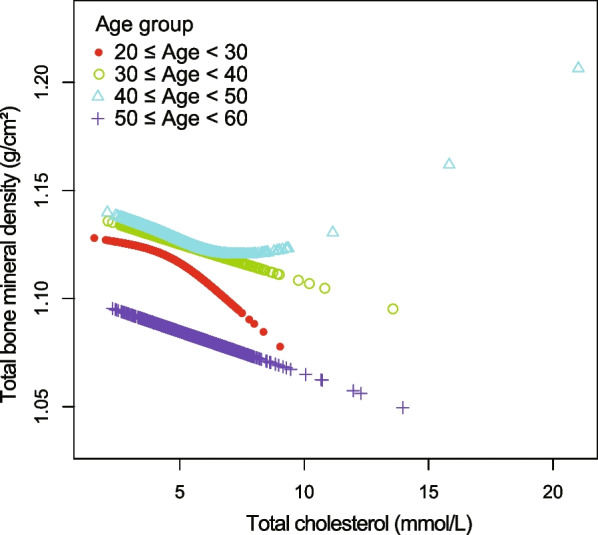
The relationship between total cholesterol and total bone mineral density stratified by age. Gender, race/ethnicity, ratio of family income-to-poverty, education level, marital status, blood urea nitrogen, serum calcium, serum phosphorus, *γ*-glutamyl transferase, ALT, AST, lactate dehydrogenase, serum uric acid, triglycerides, BMI, waist circumference, calcium intake, alcohol consumption, high blood pressure, moderate activity, diabetes, and smoking were adjusted

**Fig. 6:**
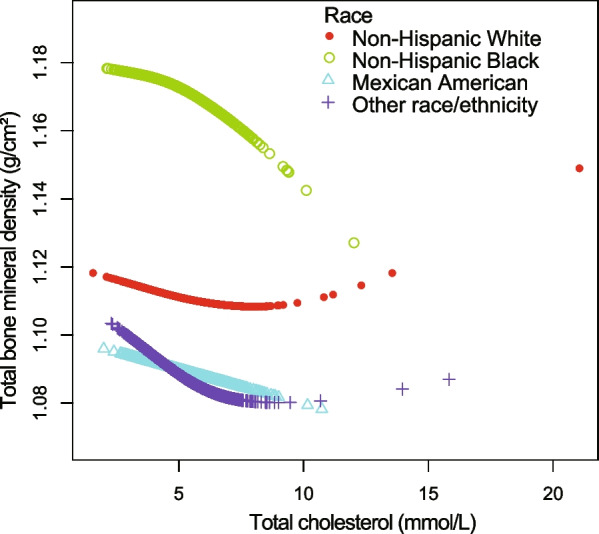
The relationship between total cholesterol and total bone mineral density stratified by race/ethnicity. Age, gender, marital status, ratio of family income-to-poverty, education level, blood urea nitrogen, serum phosphorus, γ-glutamyl transferase, ALT, AST, lactate dehydrogenase, serum uric acid, triglycerides, BMI, waist circumference, calcium intake, serum calcium, alcohol consumption, high blood pressure, moderate activity, diabetes, and smoking were adjusted

**Fig. 7:**
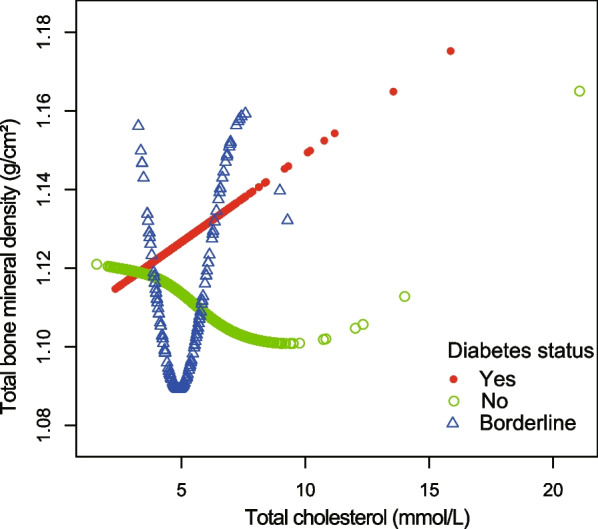
The relationship between total cholesterol and total bone mineral density stratified by diabetes. Age, gender, race/ethnicity, ratio of family income-to-poverty, education level, marital status, blood urea nitrogen, serum phosphorus, *γ*-glutamyl transferase, ALT, AST, lactate dehydrogenase, serum uric acid, triglycerides, BMI, waist circumference, calcium intake, serum calcium, alcohol consumption, high blood pressure, moderate activity, and smoking were adjusted

Figures 4, 5, 6, and 7 illustrate fitted smooth curve and generalized additive model applied to describe the linear or nonlinear relationship between total cholesterol and total BMD. In other races (including Hispanic and Multi-Racial), the relationship between total cholesterol and total BMD was a reverse-L-curve, and the inflection point was 6.7 mmol/L (Table 3). Among participants aged 40–49 years, the association between total cholesterol and total BMD was a *U*-shaped curve (Table 4). For a total cholesterol < 5.84 mmol/L, every 1 mmol/L increase in total cholesterol was associated with a 0.0063 g/cm decrease in total BMD (95%CI − 0.0117 to − 0.0009); by comparison, for individuals with a total cholesterol > 5.84 mmol/L, a 1 mmol/L increase in total cholesterol was associated with a 0.0092 g/cm increase in total BMD (95%CI 0.0017 to 0.0167). Among participants at borderline diabetes status (Table 5), a *U*-shaped curve (inflection point: 4.65 mmol/L) was presented. For total cholesterol < 4.65 mmol/L, every 1 mmol/L growth was related to a 0.0504 g/cm^2^ lower total BMD (95%CI − 0.0941 to − 0.0068); on contrast, for participants with a total cholesterol > 4.65 mmol/L, a 1 mmol/L increase in total cholesterol was linked with a 0.0293 g/cm^2^ increase in total BMD (95%CI 0.0093 to 0.0493).

**Table 3:** Threshold effect analysis of total cholesterol on total bone mineral density in other race (including Hispanic and Multi-Racial) using the two-piecewise linear regression model

Total bone mineral density	Adjusted *β* (95% CI) *P* value
Other races	
Fitting by the standard linear model	− 0.0049 (− 0.0087, − 0.0010) 0.0136
Fitting by the two-piecewise linear model	
Inflection point	6.7
Serum cholesterol < 6.7 mmol/L	− 0.0064 (− 0.0105, − 0.0023) 0.0021
Serum cholesterol > 6.7 mmol/L	0.0121 (− 0.0029, 0.0272) 0.1149
Log likelihood ratio	0.022

Age, gender, ratio of family income-to-poverty, education level, marital status, blood urea nitrogen, serum phosphorus, *γ*-glutamyl transferase, ALT, AST, lactate dehydrogenase, serum uric acid, triglycerides, BMI, waist circumference, serum calcium, calcium intake, alcohol consumption, high blood pressure, moderate activity, diabetes and smoking were adjusted

**Table 4:** Threshold effect analysis of total cholesterol on total bone mineral density in 40–49 years old group using the two-piecewise linear regression model

Total bone mineral density	Adjusted *β* (95% CI) *P* value
Aged 40–49 years old	
Fitting by the standard linear model	− 0.0006 (− 0.0045, 0.0033) 0.7696
Fitting by the two-piecewise linear model	
Inflection point	5.84
Serum cholesterol < 5.84 mmol/L	− 0.0063 (− 0.0117, − 0.0009) 0.0225
Serum cholesterol > 5.84 mmol/L	0.0092 (0.0017, 0.0167) 0.0161
Log likelihood ratio	0.003

Age, gender, race/ethnicity, ratio of family income-to-poverty, education level, marital status, blood urea nitrogen, serum phosphorus, *γ*-glutamyl transferase, ALT, AST, lactate dehydrogenase, serum uric acid, triglycerides, BMI, waist circumference, serum calcium, calcium intake, alcohol consumption, high blood pressure, moderate activity, diabetes and smoking were adjusted

**Table 5:** Threshold effect analysis of total cholesterol on total bone mineral density in borderline diabetes status using the two-piecewise linear regression model

Total bone mineral density	Adjusted *β* (95% CI) *P* value
Borderline diabetes status	
Fitting by the standard linear model	0.0087 (− 0.0055, 0.0230) 0.2295
Fitting by the two-piecewise linear model	
Inflection point	4.65
Serum cholesterol < 4.65 mmol/L	− 0.0504 (− 0.0941, − 0.0068) 0.0251
Serum cholesterol > 4.65 mmol/L	0.0293 (0.0093, 0.0493) 0.0047
Log likelihood ratio	0.003

Age, gender, race/ethnicity, ratio of family income-to-poverty, education level, marital status, blood urea nitrogen, serum phosphorus, *γ*-glutamyl transferase, ALT, AST, lactate dehydrogenase, serum uric acid, triglycerides, BMI, waist circumference, serum calcium, calcium intake, alcohol consumption, high blood pressure, moderate activity and smoking were adjusted

## Discussion

In our multivariate linear regression analyses, total cholesterol was negatively correlated with total BMD. Nevertheless, among borderline diabetes status participants, participants aged 40–49 years, and races including Hispanic and Multi-Racial, we discovered a nonlinear relationship between total cholesterol and total BMD, with inflection points 4.65 mmol/L, 5.84 mmol/L, and 6.7 mmol/L, respectively.

At present, the evidence of a relationship between total cholesterol and BMD among adults is lacking. In the past decades, epidemiological studies have shown a trend in adults that total cholesterol in plasma is increasing [26]. Age and total cholesterol concentration are widely recognized atherosclerotic cardiovascular disease (ASCVD) risk factors worldwide according to different functions that have been devised in order to provide an estimation of the possibility to bear fatal cardiovascular events [27]. But the relative influence of total cholesterol tends to be less prominent in the older adults because age itself is prevail over the other risk factors [28]. Recently, accumulated studies suggested that high cholesterol inhibits osteoblast differentiation and enhances osteoclastogenesis, thereby decreasing BMD, but the process various in different body sites [29–31]. Panagiotis Anagnostis et al. considered that serum cholesterol directly affects bone dyslipidemia via inhibiting osteoblast differentiation, accumulating in the subendothelial area of bone arteries, and activating oxidative stress in bone marrow microcirculation [32]. Among our representative US participants, a higher total cholesterol was tied with a lower total BMD in participants aged 20–59 years. Taking this relationship into account, total cholesterol may affect bone metabolism via skeletal microenvironment, and manage total cholesterol level may prevent osteoporosis or osteopenia.

Prior studies have noted the importance of total cholesterol in cardiovascular risk and osteopenia in adolescents, older people, and postmenopausal women [33, 34]. Han Hyuk Lim reported that serum total cholesterol in participants aged 10 to 18 years was significantly linked with lower BMD after adjusting for age, gender, height, and weight in Korea [35]. Joanna Makovey et al. reported that the association between total cholesterol and lumber BMD was significant negative in postmenopausal female after adjusting for age, BMI, smoke and alcohol consumption [6]. However, some follow-up research from Greece and the USA denied this negative association [36, 37]. Heterogeneity between these researches, such as different study designs, sampling methodologies, the confounding variables controlled for, and the distribution of race, may explain the debatable results. It is necessary to investigate more comprehensively the influence of total cholesterol due to high total cholesterol affecting 17.4% US adults [27].

After adjusting for covariates, our results suggested that higher total cholesterol was linked with a lower total BMD in participants aged 20–59 years in weighted multiple linear regression models. As recommended by the STROBE statement, we further performed subgroup analysis to determine a special group with diverse trends [26]. We discovered a *U*-shaped curve with an inflection point (4.65 mmol/L) between total cholesterol and total BMD in in borderline diabetes status participants. It is interesting to note that in participants aged 40–49 years, a saturation effect with an inflection point (5.84 mmol/L) was found, and a nonlinear curve for other races (including Hispanic and Multi-Racial) with an inflection point (6.7 mmol/L) was found.

In a population sample aged 20–59 years, the relationship between total cholesterol and total BMD is poorly known. As we administered a national representative sample, the findings of our study were extremely relevant to the US population. Moreover, the large sample size was feasible to conduct subgroup analyses, reporting on the association between total cholesterol and total BMD among different genders, race/ethnicity, age groups, and diabetes status. However, it is critical to acclaim the limitations in our study. First, participants with cancer or malignancy were excluded because cancer may influence the total cholesterol or total BMD, which may influence the extensibility of the conclusion. Second, the endocrine indicators like estrogen were not accessible or were absent in the NHANES 2011–2018, our findings cannot include these covariates in the present population. Third, our findings restricted the determination of causality between total cholesterol and total BMD in adults because NHANES uses a cross-sectional methodology. More bone metabolism researches and large participates prospective study is essential to comprehend the distinct mechanism of the relation among total cholesterol and total BMD.

## Data Availability

The survey data are publicly available on the interest for data users and researchers throughout the world (www.cdc.gov/nchs/nhanes/).

## Abbreviations

DEXA: Dual-energy X-ray absorptiometry
BMD: Bone mineral density
NHANES: National Health and Nutrition Examination Survey
ASCVD: Atherosclerotic cardiovascular disease
ALT: Alanine aminotransferase
AST: Aspartate aminotransferase
